# Calprotectin identified as a key differentially expressed plasma biomarker in periprosthetic joint infection against aseptic failure: a precision health proteomics study

**DOI:** 10.1186/s42836-026-00414-6

**Published:** 2026-09-04

**Authors:** Sebastian L. Andree, Linnea Salbøg Morsø, Jacob Skallerup, Bo Larsen-Ledet, Mathilde V. Søborg, Letizia Satriano, Anne C. Bay-Jensen, Sten Rasmussen, Christian F. Thudium, Allan Stensballe

**Affiliations:** 1 Department of ImmunoScience, Nordic Bioscience, Herlev, 2730 Denmark; 2 Department of Health Science and Technology, Aalborg University, Gistrup, 9260 Denmark; 3 Department of Clinical Medicine, Aalborg University, Gistrup, 9260 Denmark

**Keywords:** Periprosthetic joint infection, Calprotectin, CPa9-HNE, Arthroplasty, Biomarker, LC-MS/MS

## Abstract

**Background:**

Periprosthetic joint infection (PJI) is a debilitating and dangerous complication following joint arthroplasty procedures. With the increasing incidence of arthroplasty procedures, the burden of PJI is expected to continually grow. A correct and early diagnosis of PJI proves difficult, with the lack of a single standardized molecular test. The utilization of serology-based biomarkers would provide a non-invasive sampling method; however, these methods have not proven sufficient to satisfy the diagnostic criteria.

**Methods:**

The present study aimed to employ liquid chromatography/mass spectrometry-based (LC-MS) techniques to explore the proteomic profile of plasma samples derived from a prospective Danish PJI cohort, PRIS, to identify relevant proteins that are significantly differentially expressed in acute PJI compared to those with aseptic failure or chronic pain related to the prosthesis. Serum from patients included in the LC-MS analysis was measured for a biomarker of neutrophil activity, CPa9-HNE, measuring a neo-epitope of neutrophil elastase-cleaved calprotectin.

**Results:**

A total of 656 plasma proteins were quantified by the discovery proteomic approach, and differential abundance analysis showed increased abundance of calprotectin heterodimers, S100A8, and S100A9 in acute PJI samples compared to both aseptic failure and chronic pain. Functional enrichment of the significantly regulated proteins in acute PJI, compared to aseptic failure and chronic pain, showed an upregulation of acute immune-related pathways. Serum levels of a biomarker of neutrophil activity, an elastase-derived calprotectin neo-epitope, CPa9-HNE, showed statistically significant differences between infection and both aseptic failure and chronic pain in the same cohort, supporting the findings in LC-MS analysis.

**Conclusion:**

Plasma LC-MS proteomics and targeted measurement of CPa9-HNE neutrophil activity biomarker in matched serum samples of patients with PJI, aseptic failure, and chronic pain show significant upregulation of calprotectin heterodimers, S100A8 and S100A9, as well as CPa9-HNE in patients with PJI.

**Supplementary Information:**

The online version contains supplementary material available at https://doi.org/10.1186/s42836-026-00414-6.

## Introduction

The global demand for total knee and hip arthroplasty (TKA/THA) is steadily increasing, primarily driven by demographic aging, longer life expectancy, and the growing burden of osteoarthritis [1, 2]. By 2040, the annual counts of primary revision surgeries are projected to grow by 284% (THA) and 401% (TKA) in the United States [1]. While primary arthroplasty improves mobility and reduces pain, the growing procedural volume amplifies the burden of postoperative complications, particularly prosthetic joint infection (PJI), aseptic loosening, and chronic postsurgical pain [1, 3–5]. The global rate of PJI, based on arthroplasty registries, ranges from 0.21% to 1.45% after THA/TKA and is thought to be an underestimation, which emphasizes the growing magnitude of PJI cases [6].

PJI is a serious and resource-intensive complication of joint arthroplasty, primarily driven by biofilm-forming bacteria on implant surfaces. These biofilms, embedded in a protective extracellular matrix (ECM), shield pathogens from immune responses and antibiotic penetration, enabling persistent infection and therapeutic resistance. Their low metabolic activity and sparse planktonic bacteria reduce culture sensitivity, complicating both diagnosis and treatment [7–9].

Clinically, PJI causes pain, functional decline, prolonged antimicrobial therapy, and increased morbidity and mortality [10]. From a healthcare perspective, it imposes a substantial economic burden due to extended hospital stays and complex revision procedures, including debridement with implant retention (DAIR), one-stage, or two-stage exchange arthroplasty [10, 11].

Despite international consensus definitions, such as those proposed by the International Consensus Meeting (ICM) [12] and the European Bone and Joint Infection Society (EBJIS) [13], diagnosing PJI remains challenging. Current criteria integrate clinical, microbiological, and biochemical findings, yet no gold standard exists. Culture-negative infections and inconclusive cases are frequent, delaying treatment and complicating surgical decision-making [12, 13].

Distinguishing PJI from non-infectious conditions, such as aseptic failure and chronic pain, adds further complexity due to overlapping symptoms. Unlike PJI or loosening, chronic pain does not require revision surgery and is best managed through non-surgical approaches. Misdiagnosis may lead to unnecessary procedures, increased surgical risks, and a higher likelihood of subsequent infection. Conversely, missed infections can result in persistent disease, repeated surgeries, and poor outcomes. Accurate diagnosis is therefore essential to prevent both overtreatment and undertreatment from a precision health perspective [4, 5].

To address persistent diagnostic uncertainty, serological biomarkers have gained interest as adjunctive tools. Synovial fluid (SF) markers such as α-defensin and calprotectin show promising performance [14], but aspiration is invasive, logistically demanding, and volume may be insufficient [15, 16]. Blood-based markers (C-reactive protein (CRP), erythrocyte sedimentation rate (ESR), interleukin-6 (IL-6), fibrinogen) are widely available but lack disease specificity and are confounded by systemic inflammation, low-grade infections, prior antibiotics, and biofilm-associated infections. Consequently, many cases remain diagnostically ambiguous despite adherence to guideline algorithms [14, 17, 18].

In light of these limitations, diagnostic biomarkers with high sensitivity and specificity are urgently needed for PJI. Such markers and profiles would enable more accurate infection detection, patient stratification, and informed surgical decision-making.

High-throughput, label-free liquid chromatography-linked mass spectrometry (LC-MS) provides comprehensive plasma proteome profiling and unbiased identification of candidate biomarkers with diagnostic potential [19, 20]. This approach supports systematic discovery of proteins that could serve as robust indicators of infection, paving the way for clinical validation of implementable biomarkers. In this hypothesis-generating study, we applied proteome-wide plasma profiling using label-free quantitative proteomics on samples from the prospective PRIS arthroplasty cohort [21]. Our objective was to identify and technically confirm via ELISA blood-based proteomic profiles differentiating acute PJI from non-infectious conditions, thereby providing the groundwork for the biological validation of serum-based biomarkers.

## Methods

Blood plasma samples were obtained from patients enrolled in the multidisciplinary Prosthetic-Related-Infection and Pain (Danish acronym, PRIS) study, conducted in the North Denmark Region, with approval from the Regional Committee on Health and Research Ethics for the Northern Denmark Region (N-20110022) and the Danish Data Protection Agency (2008-58-0028). All patients included in the study had undergone TKA/THA and were referred due to prosthetic failure, defined by unexplained pain and/or mechanical issues. Diagnostic classification within the PRIS cohort comprised six categories: Acute infection, Chronic infection, Culture-negative infection, Aseptic failure, Chronic pain, and Undetermined [21].

In Khalid et al. 2020, detailing the cohort, the patients with PJI suspected within 8 weeks of the index procedure or hematogenous PJI suspected were categorized as having acute infection after postoperative microbiological confirmation, whereas if they were suspected to have PJI after 8 weeks, they were categorized as having chronic infection after postoperative microbiological confirmation [21]. Postoperative diagnosis criteria for PJI were set as three or more positive cultures with the same pathogen/s [21]. Radionuclide imaging was performed to evaluate patients with a suspected chronic pain problem [21].

The PRIS cohort was established between 2011 and 2014, before ICM2018 and EBJIS2021 diagnostic criteria. PJI confirmatory criteria in Khalid et al. 2020, of “equal to or more than three positive cultures with the same pathogen/s”, as well as the PJI-indeterminable category of “two positive cultures with the same pathogen/s”, satisfy both ICM 2018 and EBJIS 2021 criteria for infection of two or more positive cultures of the same microorganism [12, 13, 21].

### Sample preparation prior to LC-MS analysis

LC-MS-based proteomic profiling was performed using a bottom-up approach utilizing a standard protein aggregation capture (PAC) LC-MS preparatory method [22]. Blood plasma samples were digested with trypsin using an automated Opentrons OT-2 liquid handling system and analyzed by LC-MS on a timsTOF Pro2 mass spectrometer (Bruker, DE) coupled to an EvosepONE system (Evosep, DK) using 60 samples per day (60SPD) and an Evosep performance column (EV1115). Data-independent acquisition and parallel accumulation–serial fragmentation (DIA-PASEF) were employed for peptide fragmentation [23].

### Data processing

Protein identification and quantification were conducted via Spectronaut v19.5 (Biognosys, Switzerland) against the UniProt human reference proteome (May, 2025) using default settings and global normalization. The resulting protein groups data was log₂-transformed and processed in Perseus v2.1.3.0, and filtration of proteins to at least 50% valid values in at least one group, and imputation of missing values using standard parameters in Perseus simulating low abundant proteins (downshifted normal distribution with a width of 0.3 and downshift of 1.8) for the principal component analysis (PCA), differential abundance analysis, and downstream functional enrichment [24]. Three samples were found to have been collected in two tubes each and thus measured twice by LC-MS; these duplicate measurements were then averaged together.

### Statistical data analysis

Differential abundance analysis was performed using two Welch’s t-tests, and the *p*-values were false discovery rate (FDR) corrected via default permutation settings in Perseus [24, 25]. An FDR-corrected *p*-value cutoff of < 0.05 indicated statistical significance for downstream analysis and visualization. PCA visualization was conducted within Perseus [24].

For the unsupervised PCA, all diagnostic groups were included to explore global proteomic variation across the cohort. In subsequent differential abundance analyses, including volcano plots and bioinformatic network analysis, only patients with a final diagnosis of Acute infection were retained in the PJI group. Patients categorized with Chronic infection, Culture-negative infection, and Undetermined were excluded due to low sample counts of *n* = 2, *n* = 1, and *n* = 4, respectively.

Matrices derived from the t-tests were exported, and results were visualized in volcano plots created in RStudio v2026.1.1.403 with ggplot2 v3.5.2 in tidyverse v2.0.0 and cowplot v1.1.3 in R v4.4.0 with attached packages [26–30]. Bioinformatic network analysis of significantly regulated proteins in the Infection vs Chronic pain group was conducted using the STRING webapp v12.0 [31]. The Human Plasma Peptide Atlas Full 2025-08 Build was downloaded and utilized as the statistical background in STRING [32]. Proteins in the significantly regulated proteins uploaded into STRING that were not recognized by STRING, mostly immunoglobulin-related proteins, were kicked out of the analysis. Functional enrichment analysis was conducted in STRING, and the top pathways represented and sorted by FDR for Gene Ontology Biological Process were utilized. For additional exploratory analysis of upregulated proteins and pathways in acute infection against a larger group of non-infected samples, the Aseptic failure and Chronic pain groups were combined into the “Sterile” group; this does not imply shared biological pathology between these combined groups.

### CPa9-HNE biomarker measurement and analysis

CPa9-HNE, developed and provided by Nordic Bioscience A/S, was measured by a competitive enzyme-linked immunosorbent assay (ELISA) in available PRIS cohort serum samples matching the patients whose plasma was analyzed via LC-MS in Table 1, *n* = 77: Aseptic failure (*n* = 41), Chronic pain (*n* = 18), and Infection (*n* = 18). The assay was conducted as described previously [33].

**Table 1:** Clinical parameters of the subset of the PRIS cohort plasma samples investigated by quantitative LC-MS proteomics

Characteristic	Total patients (*N* = 84)
**Age, years**	
Median, Interquartile range (IQR)	71 (66–78)
Range	39–93
**Sex, *n* (%)**	
Female	37 (44.0%)
Male	47 (56.0%)
**Type of Arthroplasty, *n* (%)**	
Total Knee Arthroplasty (TKA)	40 (47.6%)
Total Hip Arthroplasty (THA)	44 (52.4%)
**Comorbidities, *n* (%)**	
Cardiovascular disease	23 (27.4%)
Diabetes mellitus (Type 1 or 2)	11 (13.1%)
Malignancy*	9 (10.7%)
Rheumatoid disease/inflammatory condition*	8 (9.5%)
Biological immunotherapy	8 (9.5%)
Chronic obstructive pulmonary disease (COPD)	1 (1.2%)
**Diagnosis after PRIS, *n* (%)**	
Aseptic loosening	41 (48.8%)
Acute infection	18 (21.4%)
Chronic pain	18 (21.4%)
Undetermined	4 (4.8%)
Chronic infection	2 (2.4%)
Culture-negative infection	1 (1.2%)

Bold lettering indicates categories of clinical parameters within the table. *Data were missing for one patient in each of the cancer and rheumatoid disease/inflammatory condition categories (two distinct individuals). One patient categorized as a chronic infection was additionally previously categorized as an acute infection as a separate donor

Raw data of the CPa9-HNE measurement were statistically processed in R with rstatix v0.7.2 and tidyverse packages utilizing non-parametric tests in RStudio [26, 28, 30, 34]. A Kruskal–Wallis test was first used before further statistical evaluation with the Dunn test corrected for multiple comparisons with the Holm method. Data were visualized in R and RStudio, with ggplot2 v3.5.2 and ggforce v0.5.0. Statistical bars were added manually in accordance with results from R [26, 27, 30, 35]. A multivariable linear model analysis was performed to include patients with rheumatoid disease/inflammatory condition and patients receiving biological immunotherapy in R, utilizing ln-transformed CPa9-HNE concentrations to create a normal distribution [26, 30]. The adjusted model, including the patients with the aforementioned comorbidities, is compared to the unadjusted model (Supplemental Table S1). Both models utilize Infection as the reference group against Aseptic failure and Chronic pain without correction of *p*-values for multiple hypothesis testing. One patient was removed in both models due to an NA value in the rheumatoid disease/inflammatory condition. The linear model showed minimal effect of these comorbidities, and the results are shown in Supplemental Table S1. Microsoft PowerPoint v2604 was used to refine figures.

### Availability of data and materials

The proteomic dataset supporting the conclusions of this article are available from the public ProteomXchange consortia under the number (PXD071394).

## Results

**Fig. 1:**
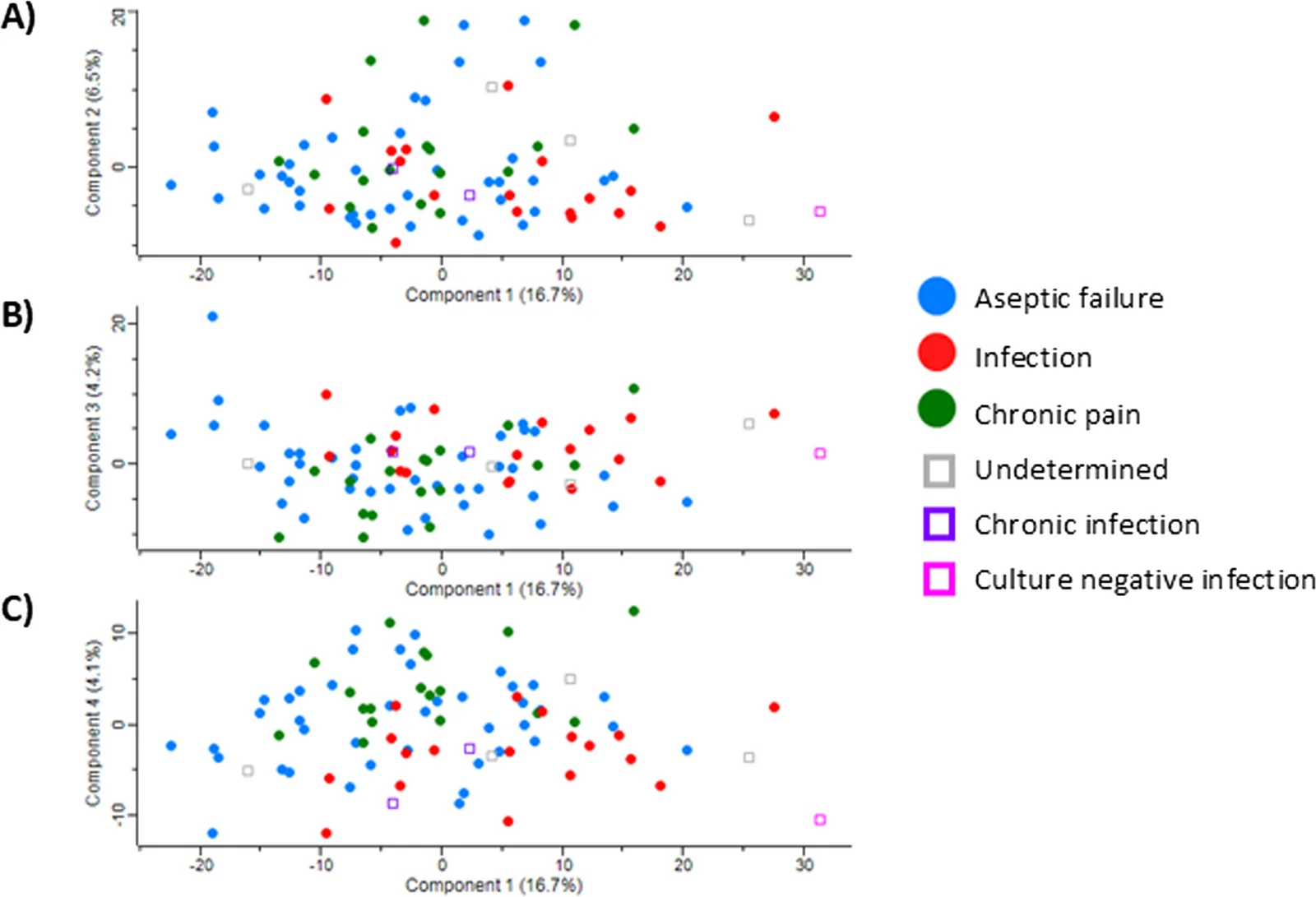
Principal component analysis (PCA) of the first three PCs showing the variance explained by each with the combination of (**A**) PC1 vs PC2, (**B**) PC1 vs PC3, and (**C**) PC1 vs PC4

A PCA was done to analyze the separation of the different groups based on their LC-MS-derived proteomic intensity signatures and their similarities (Fig. 1). 16.7% of the variation in the data is explained by PC1, 6.5% by PC2, 4.2% by PC3, and 4.1% by PC4. Despite the highest explanation of variance in PC1 and PC2, none of the groups were clearly separated on these PCs. When visualized against PC1, PC3, and PC4 did, however, start to show a visual separation between the Chronic pain group and Infection, highlighting observational differences between these groups based on the PC loadings alone (Fig. 1B, C).

**Fig. 2:**
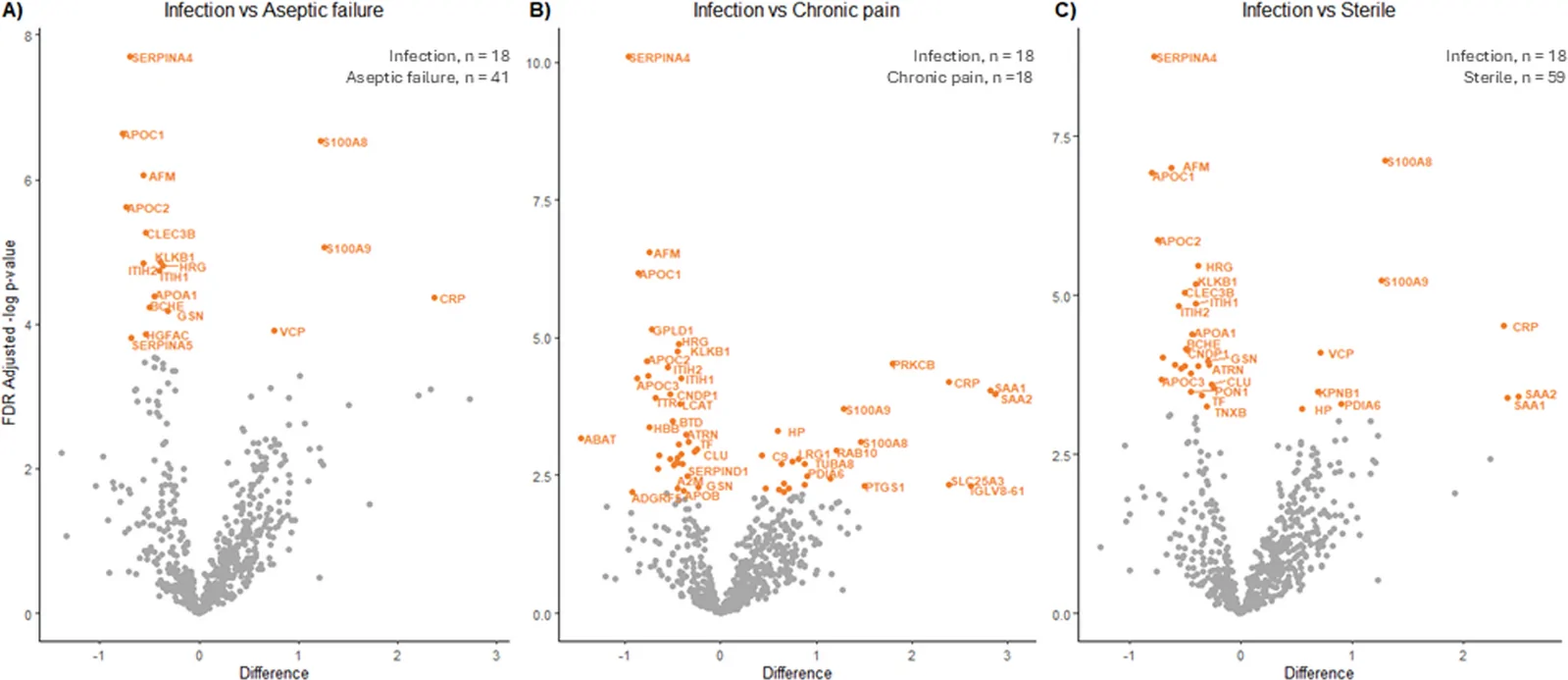
Volcano plots highlighting significant (FDR-adjusted *p*-value < 0.05) differentially abundant protein gene names in orange between test conditions: (**A**) Infection vs Aseptic failure, (**B**) Infection vs Chronic pain, and (**C**) Infection vs Sterile. Gray points represent differentially abundant genes with no significance. A positive difference indicates the upregulated proteins of the first comparison group in the subheading against the second comparison group, whereas a negative difference indicates the downregulated proteins of the first comparison group in the subheading against the second comparison group

In evaluating the regulated proteins discovered by the LC-MS, the bottom-up proteomic analysis yielded 675 identified proteins from the whole sample range. After strict filtration of the data, a total of 656 proteins were retained. Differential abundance analysis between the three main groups, Infection, Aseptic failure, and Chronic pain, as well as subsequent pooled Sterile group containing the Aseptic failure and Chronic pain groups, revealed significant proteins under an FDR-adjusted *p*-value of 0.05 in all comparisons in Fig. 2. Comparing Infection and Aseptic failure (Fig. 2A), both proteins of the calprotectin heterodimer [36], S100A8 and S100A9 were the most significantly upregulated in Infection, with S100A8 being the most significant. CRP and VCP (transitional endoplasmic reticulum ATPase) were also significantly upregulated in Infection. Comparing Infection vs. Chronic pain (Fig. 2B) showed a high quantity of significantly different proteins on both sides. 25 proteins were overabundant, and 36 proteins were underabundant in Infection. Immune-related acute-phase proteins, such as CRP, SAA1 (serum amyloid A1), and SAA2 (serum amyloid A2), and calprotectin, were found significantly abundant in the Infection group (Fig. 2B). The comparison between Chronic pain and Aseptic failure did not reveal any significant differences (Supplementary Fig. S1). To demonstrate that imputation did not affect the resulting significantly upregulated proteins in Infection vs Aseptic failure and Chronic pain, a differential expression analysis was performed on these groups with unimputed data (Supplementary Fig. S3). Additionally, a differential expression sensitivity analysis excluding patients with rheumatoid disease/inflammatory condition and/or patients receiving biological immunotherapy was performed, showing that these conditions have little effect on the significantly upregulated proteins in Infection in these comparisons (Supplementary Fig. S4). To further investigate significantly differentially abundant proteins between acute infection and non-infected patients, Aseptic failure and Chronic pain, two non-infected groups, were pooled together in a group termed ‘Sterile’ for additional exploratory comparison against Infection (Fig. 2C). In the Infection vs Sterile groups comparison, the calprotectin complex (S100A8 and S100A9) remained the most significantly upregulated protein.

**Fig. 3:**
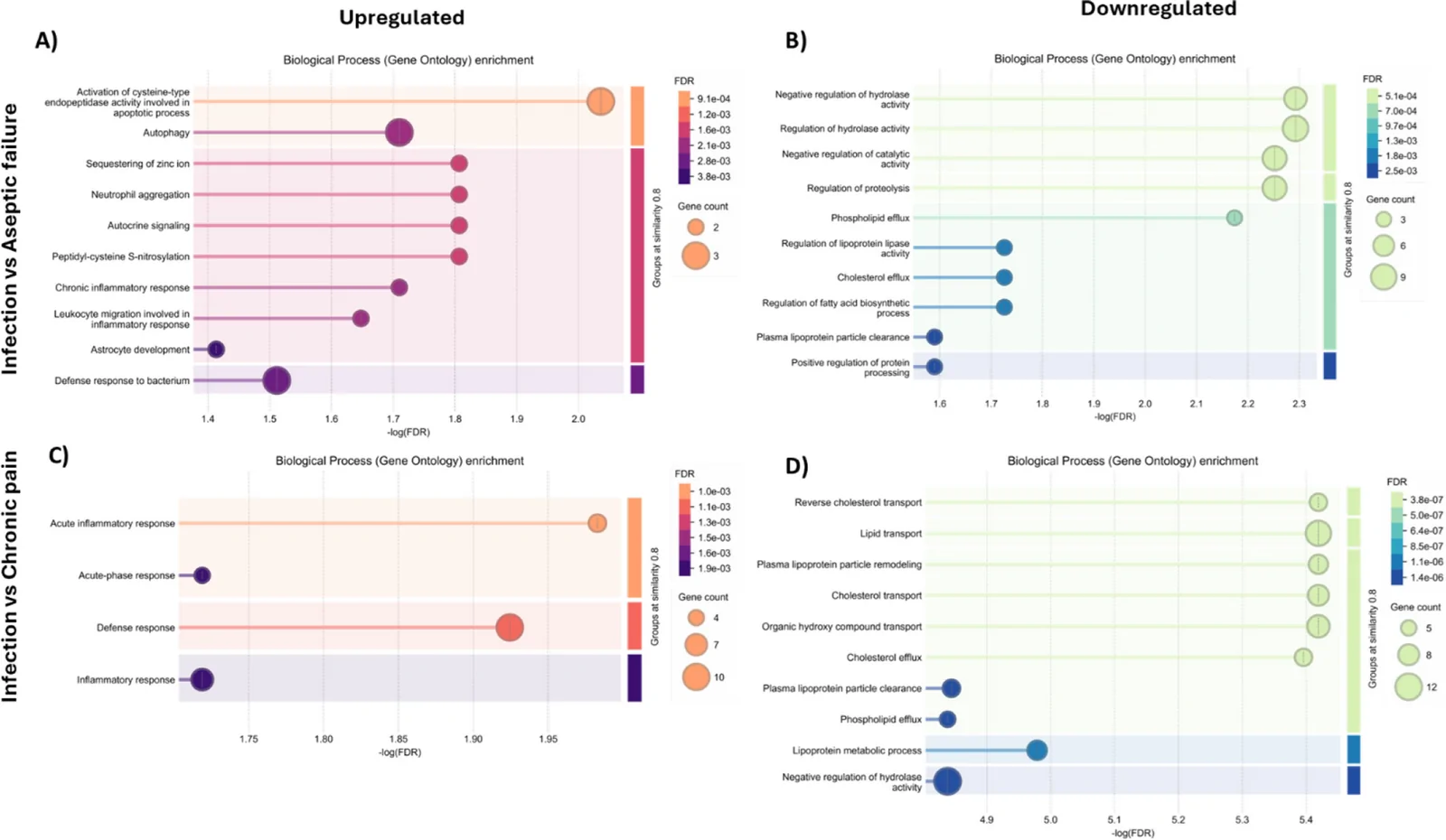
Functional enrichment analysis (**A–D**) of significantly upregulated (**A&C**) and downregulated (**B&D**) proteins in Infection vs. Chronic pain, sorted by FDR

To explore how the molecular pathways differ in the Infection group compared to the Chronic pain and Aseptic failure, a functional enrichment analysis was conducted on the significantly up- and down-regulated proteins found in Fig. 2A and B. The top Biological Process Gene Ontology pathways involved in both the upregulated (Fig. 3A, C) and downregulated (Fig. 3B, D) fractions of the Infected group versus Chronic Pain and Aseptic failure are depicted. As expected, in the upregulated fractions, the acute inflammatory response and defense response prevailed as the top pathways against Chronic pain, whereas the more specific neutrophil-related pathways were dysregulated in Infection against Aseptic failure, likely due to the lower number of significantly different proteins (Fig. 3A, C). In the downregulated fractions of Infection against Aseptic failure and Infection against Chronic pain, hydrolase activity and reverse cholesterol transport, respectively, dominate as top pathways (Fig. 3B, D). Functional enrichment of the Infection vs Aseptic failure group is attached as Supplemental Fig. S2, showing acute inflammatory response as the most significant pathway in Infection.

**Fig. 4:**
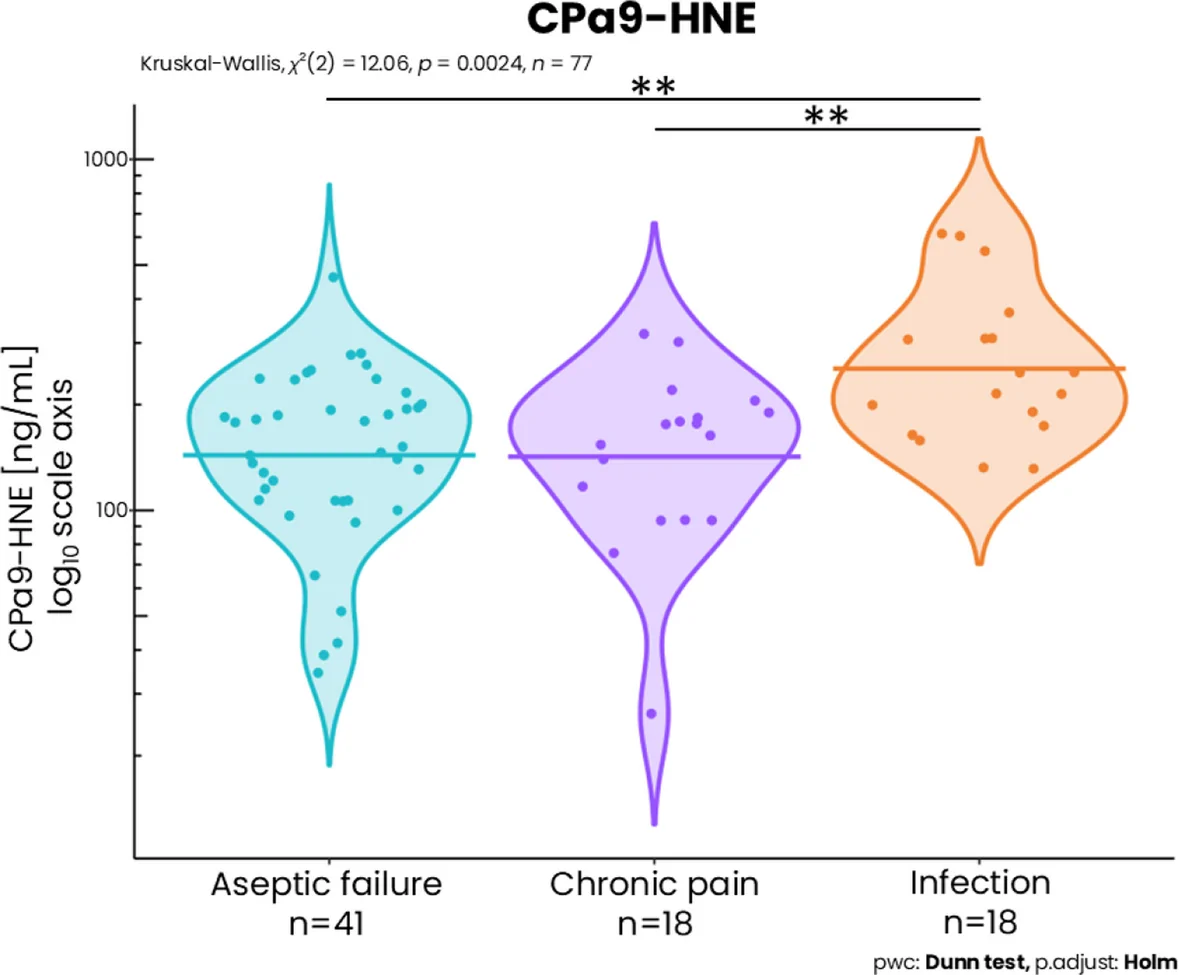
CPa9-HNE biomarker measurement in matched serum samples in the PRIS cohort. **Represents a significance threshold of *p* < 0.01. The crossbar represents the group means

To further investigate the upregulation of calprotectin in the Infection group, a targeted ELISA-based method was employed in available matching serum of the same patients analyzed by LC-MS to measure a biomarker of neutrophil activity, CPa9-HNE, measuring a neo-epitope of S100A9 generated via cleavage by neutrophil elastase. CPa9-HNE levels were significantly elevated in Infection compared to Aseptic failure and Chronic pain Fig. 4. These results support elevated neutrophil activity in infection, consistent with the differential abundance analysis and functional enrichment analysis. No difference was observed between Aseptic failure and Chronic pain, which is likewise consistent with the differential abundance analysis (Fig. 4 & Supplemental Fig. S1).

## Discussion

This study of patients with prosthetic joint complications unveils important systemic inflammatory protein signatures and biological pathways that characterize acute PJI and post-surgical chronic pain through deep plasma precision proteomic methods. Additionally, we utilize a promising serum neutrophil activity biomarker ELISA, CPa9-HNE, in available matching serum of the same patients to technically confirm the findings of immune-related proteins and pathways from LC-MS analyses. It is important to note that findings concerning the Infection group are limited to acute PJI, as the analyzed cohort only contained enough of these patients for meaningful analysis. Our findings contribute to the ongoing efforts to profile PJI through unbiased proteomic techniques with LC-MS. Through this data, we also investigate the relative abundance of both established and emerging biomarkers for PJI, such as CRP and calprotectin, within blood plasma [12, 37].

Currently, LC-MS is not widely implemented for routine clinical diagnostics of patient samples at a large scale in blood plasma analysis, likely due to its high cost and complex technical requirements for operation and data analysis [38]. However, its utility in biomarker discovery for future translational clinical applications is evident, and workflows for utilizing LC-MS-proteomics in larger clinical studies have been optimized for this purpose [39, 40]. Translation of biomarkers onto platforms such as ELISA-based techniques allows for accessible and streamlined implementation in clinical settings [41]. To the best of our knowledge, this is the first study to profile the proteomic landscape of PJI within plasma and thus provides novel insight into systemic indications of the disease.

Calprotectin, a heterodimer composed of S100A8 and S100A9, is key to fecal diagnostics of IBD but has now gained increasing attention as a diagnostic marker in PJI, particularly in synovial fluid, with multiple studies demonstrating the high sensitivity and specificity for detecting PJI in joint aspirates [42–44]. Furthermore, synovial calprotectin has recently received support at the ICM 2025 as a diagnostic, with a ‘Strong’ level of evidence [45]. Although synovial fluid or stool remains the primary matrix for calprotectin testing, there is growing evidence of its application in blood [43, 44]. Blood sampling offers practical advantages over joint aspiration, as the latter is invasive and can be limited by insufficient fluid volume, and has postoperative changes in the synovial fluid composition [46–48]. Collectively, these findings support the growing interest in calprotectin as a potential diagnostic biomarker for PJI, particularly in blood-based assays. Higher sensitivity/specificity of both blood (93.3% & 87.5%) and SF (95.6% & 95%) calprotectin in comparison to blood (80% & 87.5%) and SF (64.4% and 95%) CRP additionally point towards calprotectin as being a more effective diagnostic biomarker [43].

Consistent with these findings, our differential abundance analysis of plasma from patients with and without acute PJI revealed that calprotectin subunits emerged as the most consistently upregulated proteins in patients with acute infection, further highlighting the role of calprotectin in infection-specific immune responses and is observable on a systemic, blood-based level.

The present study also demonstrates that a calprotectin neo-epitope generated by neutrophil elastase cleavage, measured by CPa9-HNE, is significantly increased in blood from infected patients, providing support as a derivative of calprotectin that blood-based calprotectin may be able to provide discriminatory potential in differentiating acute PJI from non-infectious complications, including aseptic failure and chronic pain, through further clinical evaluation.

CPa9-HNE measures neutrophil activity through this interaction of calprotectin and neutrophil elastase, which are both released upon activation of the neutrophils and have been shown to measure a greater fold change between activated neutrophils and inactive neutrophils of 101-fold in contrast to measurements of full-length calprotectin, MRP8/14, of 0.6-fold [33]. Preliminary testing of CPa9-HNE in the serum of the patients analyzed with LC-MS in the present study shows that the high levels of CPa9-HNE in Infection indicate that neutrophil activity is increased as well. This increased neutrophil activity supports the enrichment findings of increased immune activity in the Infection group compared to Aseptic failure and Chronic pain. Of note, this is the first study to utilize the CPa9-HNE biomarker to evaluate neutrophil activity within the context of PJI. The EBJIS diagnostic definition utilizes neutrophil counts in histological evaluations; however, neutrophil activity itself, uncoupled from counts or percentage, is not currently used as a diagnostic determinant [13]. It should also be noted that due to the authors’ commercial affiliations to Nordic Bioscience A/S, the developer and producer of CPa9-HNE, the preliminary findings require independent third-party validation in future cohorts.

The functional enrichment analysis of the significantly differentially expressed proteins between the Infection and Chronic pain groups characterizes biological processes that phenotypically separate the two patient groups. In the Infection group, gene ontology terms are dominated by acute inflammatory, acute phase, and defense responses. The upregulated profile of Infection against Aseptic failure seems to be more pathway-specific, such as the activation of protease activity and metal ion sequestration. This may be due to the low number of proteins present in the upregulated fraction and calprotectin’s presence therein. Comparison of Infection against the Sterile pooled group in Fig. 2C and Supplemental Fig. S2 was done for exploratory purposes to compare the Infection group against these groups, which do not have infection, but does not assume a greater biological similarity beyond this.

In contrast, both Aseptic failure and Chronic pain groups are characterized by enrichment of biological processes mainly linked to cholesterol-associated mechanisms and plasma lipoprotein remodeling. These findings suggest a distinct biological profile of metabolic regulation downregulated in the Infection group. In a study by Mi et al., the authors conclude that high-density lipoprotein cholesterol (HDL-C) is negatively correlated with chronic pain; however, proteins upregulated in patients with chronic pain compared to infection consist heavily of HDL components [49].

Acute phase proteins CRP, SAA1, and SAA2 were also upregulated in Infection against patients with chronic pain, with CRP being concurrently upregulated in Infection against Aseptic failure. CRP is a widely used biomarker for inflammation and infection and represents one of the few serum-based diagnostic markers included in the disease scoring system of the 2018 definition of PJI [12]. SAA is investigated in the context of acute phase biomarkers in inflammatory rheumatic diseases and increases and decreases rapidly as a reactant [50]. SAA has been measured in serum in the context of PJI, and the authors found a sensitivity of 85% and specificity of 96% for PJI compared to non-PJI patients who underwent revision THA/TKA [51]. Interestingly, in the present study, SAA1 or SAA2 was not found to be significant in Infection against Aseptic failure despite being significant in Infection vs Chronic pain, perhaps due to chronic inflammation being a pathogenic driver of aseptic loosening [52, 53].

The presence of confounding comorbidities, often found in older patients [54], may pose risks to the utilization of broad inflammation markers [55]. Within the PJI field, there is still limited evidence to conclude whether or not these comorbidities affect diagnosis outcomes with serum or synovial biomarkers due to divergent study outcomes [56]. This uncertainty may indicate that systemic inflammation markers, such as CRP [50, 57], may thus be a suboptimal clinical marker for PJI. Nevertheless, the upregulation in Infection against Aseptic failure and Chronic pain may still provide diagnostic value to differentiate these groups from infection with further validation. The diagnostic ambiguity of pain has been commonly commented on within the field, with the mere presence of pain leading to suspicion of a potential PJI [13, 58]. Consequently, biomarkers for PJI must be able not only to separate infection from Aseptic failure, but also to distinguish from patients experiencing chronic pain. From the LC-MS data, there was no significant difference between the Chronic pain group and Aseptic failure, which suggests that finding these biomarkers to separate between these conditions may be difficult; however, separating acute infection from these aseptic conditions shows promise.

Together, the differential abundance analysis, functional enrichment analysis, and CPa9-HNE measurements underscore the importance of immune-related processes in separating acute PJI from non-infectious postoperative pain and aseptic failure. From the PCA, we can see that the first two PC do not separate any of the sample groups, thus showing that a large heterogeneity between groups in the proteomic context of aseptic failure, chronic pain, and infection exists, further showing the need for key targeted protein biomarkers. The consistent upregulation of acute inflammatory and phase responses highlights a solid innate immune signature in patients with acute infection following total joint arthroplasty (TJA). These findings collectively suggest that targeting immune-related biomarkers, such as calprotectin, is of diagnostic and biological relevance to discriminate between revision-requiring cases of acute PJI and cases of chronic pain. Accurate stratification of patient groups holds great clinical interest and may help reduce unnecessary surgical procedures and exposure to inappropriate risks in patients experiencing postoperative chronic pain [4].

Due to the exploratory nature of this study, several limitations of the cohort and methods limit the scope of conclusions. The small sample size available for analysis of *n* = 18 for both Chronic pain and Infection, and *n* = 41 for Aseptic failure, is unable to capture the true heterogeneity of PJI. Because of this, a major limitation of these findings is the lack of an independent external validation cohort, which is also required due to the authors’ commercial affiliations with Nordic Bioscience A/S. Additionally, clinical information regarding the microbial profile of infected patients was unavailable during the analysis, making it impossible to segment the proteomes via underlying pathogen profile. Intrinsic limitations of the methods utilized for analysis of this cohort also exist, such as the imputation of LC-MS intensity data utilizing a downshifted normal distribution, which may artificially compress variance and inflate statistical significance in small cohorts, although sensitivity differential expression analysis of unimputed data shows a minor effect on the top significantly regulated proteins.

## Conclusion

We successfully employed a deep LC-MS-based plasma proteomics profiling of a clinical PJI cohort, revealing calprotectin, an increasingly promising diagnostic biomarker, as the most significantly abundant protein in acute PJI against aseptic failure. Pathways associated with the innate immune system heavily characterize the systemic proteome of localized infection and biologically support the use of immune-related biomarkers for the diagnosis of acute PJI. Additionally, the serum biomarker, CPa9-HNE, measuring neutrophil activity, technically confirms the proteomic findings derived from the LC-MS analysis by showing a significant increase in neutrophil activity in the Infection group. Due to the sample composition of the study, interpretation of the data is limited to acute PJI, thus requiring further profiling of chronic PJIs. Continued investigation of the upregulated proteins in PJI, in particular calprotectin, may show promise as systemic, blood-derived biomarkers in the diagnosis of PJI with further clinical validation.

## Supplementary Information

Supplementary Material 1.

## Data Availability

The proteomic dataset supporting the conclusions of this article is available via the ProteomeXchange Consortium under accession number PXD071394.

## Abbreviations

PJI: Periprosthetic joint infection
LC-MS: Liquid chromatography coupled/mass spectrometry
TKA: Total knee arthroplasty
THA: Total hip arthroplasty
ECM: Extracellular matrix
DAIR: Debridement, antibiotics, irrigation, and retention
ICM: International consensus meeting
EBJIS: European Bone and Joint Infection Society
SF: Synovial fluid
CRP: C-reactive protein
ESR: Erythrocyte sedimentation rate
IL-6: Interleukin 6
PRIS: Prosthetic-related-infection and pain [cohort]
PAC: Protein aggregation capture
60SPD: 60 samples per day
DIA-PASEF: Data-independent acquisition and parallel accumulation–serial fragmentation
PCA: Principal component analysis
FDR: False discovery rate
ELISA: Enzyme-linked immunosorbent assay
IQR: Interquartile range
COPD: Chronic obstructive pulmonary disease
VCP: Transitional endoplasmic reticulum ATPase
SAA1: Serum amyloid A1
SAA2: Serum amyloid A2
HDL-C: High-density lipoprotein cholesterol
TJA: Total joint arthroplasty

## References

[1] Singh JA, Yu S, Chen L, Cleveland JD. Rates of total joint replacement in the United States: Future projections to 2020–2040 using the national inpatient sample. J Rheumatol. 2019;46(9):1134–40. 30988126 10.3899/jrheum.170990

[2] Stubnya BG, Schulz M, Váncsa S, Szilágyi GS, Szatmári A, Bejek Z. Global Trends in Joint Arthroplasty : A Systematic Review and Future Projections. J Clin Med. 2025;14(22):1–18. 10.3390/jcm14228214 PMC12653837 41303249

[3] Heo SM, Harris I, Naylor J, Lewin AM. Complications to 6 months following total hip or knee arthroplasty: Observations from an Australian clinical outcomes registry. BMC Musculoskelet Disord. 2020;21(602):1–11. 10.1186/s12891-020-03612-8 PMC7488141 32912197

[4] Kelmer G, Stone AH, Turcotte J, King PJ. Reasons for Revision: Primary Total Hip Arthroplasty Mechanisms of Failure. J Am Acad Orthop Surg. 2021;29(2):78–87. 32404682 10.5435/JAAOS-D-19-00860

[5] Petersen KK, Simonsen O, Laursen MB, Nielsen TA, Rasmussen S, Arendt-Nielsen L. Chronic postoperative pain after primary and revision total knee arthroplasty. Clin J Pain. 2015;31(1):1–6. 25485953 10.1097/AJP.0000000000000146

[6] Piuzzi NS, Sigmund IK, Slullitel PA, Budhiparama N, Manning L, Visperas A, et al. Global Perspectives on the Management of Periprosthetic Joint Infection. J Bone Jt Surg. 2025;107(22):2521–8. 10.2106/JBJS.25.00775 41315038

[7] Staats A, Li D, Sullivan AC, Stoodley P. Biofilm formation in periprosthetic joint infections. Ann Jt. 2021;6(43):1–10. 10.21037/aoj-20-85 PMC8635410 34859164

[8] Arnold W V., Shirtliff ME, Stoodley P. Bacterial biofilms and periprosthetic infections. Vol. 95(24), The Journal of Bone and Joint Surgery. 2013. p. 2223–9. 10.2106/JBJS.2223 PMC6948784 24498639

[9] Piuzzi NS, Klika AK, Lu Q, Higuera-Rueda CA, Stappenbeck T, Visperas A. Periprosthetic joint infection and immunity: Current understanding of host–microbe interplay. J Orthop Res. 2025;42(1):7–20. 10.1002/jor.25723 37874328

[10] Xu Y, Huang TB, Schuetz MA, Choong PFM, the ICARUS group. Mortality, patient-reported outcome measures, and the health economic burden of prosthetic joint infection. EFORT Open Rev. 2023;8(9):690–7. 10.1530/EOR-23-0078 PMC10548306 37655835

[11] Alt V, Szymski D, Rupp M, Fontalis A, Vaznaisiene D, Marais LC, et al. The health-economic burden of hip and knee periprosthetic joint infections in Europe a comprehensive analysis following primary arthroplasty. Bone Jt Open. 2025;6(3):298–311. 40054494 10.1302/2633-1462.63.BJO-2024-0225.R1 PMC11888791

[12] Parvizi J, Tan TL, Goswami K, Higuera C, Della Valle C, Chen AF, et al. The 2018 Definition of Periprosthetic Hip and Knee Infection: An Evidence-Based and Validated Criteria. J Arthroplasty. 2018;33(5):1309-1314.e2. 29551303 10.1016/j.arth.2018.02.078

[13] McNally M, Sousa R, Wouthuyzen-Bakker M, Chen AF, Soriano A, Vogely HC, et al. The EBJIS definition of periprosthetic joint infection: A practical guide for clinicians. Bone Jt J. 2021;103-B(1):18–25. 10.1302/0301-620X.103B1.BJJ-2020-2417 PMC7954145 33380197

[14] Yilmaz MK, Abbaszadeh A, Tarabichi S, Azboy I, Parvizi J. Diagnosis of periprosthetic joint infection: the utility of biomarkers in 2023. Antibiotics (Basel). 2023;12(6, 1054):1–16. 10.3390/antibiotics12061054 PMC10295740 37370373

[15] Matsen Ko L, Parvizi J. Diagnosis of Periprosthetic Infection. Novel Developments Orthop Clin North Am. 2016;47(1):1–9. 26614915 10.1016/j.ocl.2015.08.003

[16] Zahar A, Lausmann C, Cavalheiro C, Dhamangaonkar AC, Bonanzinga T, Gehrke T, et al. How reliable is the cell count analysis in the diagnosis of prosthetic joint infection? J Arthroplasty. 2018;33(10):3257–62. 29887359 10.1016/j.arth.2018.05.018

[17] Tripathi S, Tarabichi S, Parvizi J, Rajgopal A. Current relevance of biomarkers in diagnosis of periprosthetic joint infection: an update. Arthroplasty. 2023;5(41):1–10. 37525262 10.1186/s42836-023-00192-5 PMC10391917

[18] Akcaalan S, Ozaslan HI, Caglar C, Şimşek ME, Citak M, Akkaya M. Role of Biomarkers in Periprosthetic Joint Infections. Diagnostics. 2022;12(12, 2958):1–11. 10.3390/diagnostics12122958 PMC9777153 36552965

[19] Woo J, Zhang Q. A Streamlined High-Throughput Plasma Proteomics Platform for Clinical Proteomics with Improved Proteome Coverage, Reproducibility, and Robustness. Journa Am Soc Mass Spectrom. 2023;34(4):754–62. 10.1021/jasms.3c00022 PMC10080683 36975161

[20] Distler U, Yoo HB, Kardell O, Hein D, Sielaff M, Scherer M, et al. Multicenter evaluation of label-free quantification in human plasma on a high dynamic range benchmark set. Nat Commun. 2025;16(8774):1–18. 41038884 10.1038/s41467-025-64501-z PMC12491457

[21] Khalid V, Schønheyder HC, Larsen LH, Nielsen PT, Kappel A, Thomsen TR, et al. Multidisciplinary diagnostic algorithm for evaluation of patients presenting with a prosthetic problem in the hip or knee: a prospective study. Diagnostics (Basel). 2020;10(2, 98):1–14. 10.3390/diagnostics10020098 PMC7168188 32053936

[22] Batth TS, Tollenaere MAX, Ru P, Gonzalez-franquesa A, Batth TS, Tollenaere MAX, et al. Protein Aggregation Capture on Microparticles Enables Multipurpose Proteomics Sample Authors Protein Aggregation Capture on Microparticles Enables Multipurpose Proteomics Sample. 2019;(May):1027–35. 10.1074/mcp.TIR118.001270 PMC6495262 30833379

[23] Meier F, Brunner AD, Frank M, Ha A, Bludau I, Voytik E, et al. diaPASEF: parallel accumulation–serial fragmentation combined with data-independent acquisition. Nat Methods. 2020;17:1229–36. 10.1038/s41592-020-00998-0 33257825

[24] Tyanova S, Temu T, Sinitcyn P, Carlson A, Hein MY, Geiger T, et al. The Perseus computational platform for comprehensive analysis of (prote)omics data. Nat Methods. 2016;13(9):731–40. 27348712 10.1038/nmeth.3901

[25] Shuken SR, McNerney MW. Costs and Benefits of Popular P-Value Correction Methods in Three Models of Quantitative Omic Experiments. Anal Chem. 2023;95(5):2732–40. 36693222 10.1021/acs.analchem.2c03719 PMC10653731

[26] Posit team. RStudio: Integrated Development Environment for R. Boston, MA: Posit Software, PBC; 2025. Available from: http://www.posit.co/

[27] Wickham H. Ggplot2: elegant graphics for data analysis. New York: Springer-Verlag; 2016.

[28] Wickham H, Averick M, Bryan J, Chang W, Mcgowan LDA, François R, et al. Welcome to the Tidyverse. 2019;4:1–6.

[29] Wilke CO. cowplot: Streamlined Plot Theme and Plot Annotations for “ggplot2”. 2024. Available from: https://cran.r-project.org/package=cowplot

[30] R Core Team. R: A Language and Environment for Statistical Computing. R Foundation for Statistical Computing, Vienna, Austria; 2024. Available from: https://www.r-project.org/

[31] Szklarczyk D, Kirsch R, Koutrouli M, Nastou K, Mehryary F, Hachilif R, et al. The STRING database in 2023: protein-protein association networks and functional enrichment analyses for any sequenced genome of interest. Nucleic Acids Res. 2023;51(D1):D638–46. 36370105 10.1093/nar/gkac1000 PMC9825434

[32] Deutsch EW, Omenn GS, Sun Z, Maes M, Pernemalm M, Palaniappan KK, et al. Advances and Utility of the Human Plasma Proteome. J Proteome Res. 2021;20(12):5241–63. 34672606 10.1021/acs.jproteome.1c00657 PMC9469506

[33] Mortensen JH, Sinkeviciute D, Manon-Jensen T, Domislović V, McCall K, Thudium CS, et al. A Specific Calprotectin Neo-epitope [CPa9-HNE] in Serum from Inflammatory Bowel Disease Patients Is Associated with Neutrophil Activity and Endoscopic Severity. J Crohn’s Colitis. 2022;16(9):1447–60. 35304895 10.1093/ecco-jcc/jjac047 PMC9455793

[34] Kassambara A. rstatix: Pipe-Friendly Framework for Basic Statistical Tests. 2023. Available from: https://cran.r-project.org/package=rstatix

[35] Pedersen TL. ggforce: Accelerating “ggplot2”. 2025. Available from: https://ggforce.data-imaginist.com/

[36] Jukic A, Bakiri L, Wagner EF, Tilg H, Adolph TE. Calprotectin: from biomarker to biological function. Gut. 2021;70(10):1978–88. 34145045 10.1136/gutjnl-2021-324855 PMC8458070

[37] Magruder ML, Heckmann ND, Lieberman JR, Scuderi G, Lustig S, Parvizi J, et al. Novel Technologies in Periprosthetic Joint Infection: Emerging Diagnostics. J Arthroplasty. 2025;41(2):551–9. 10.1016/j.arth.2025.08.002 40782944

[38] Vogeser M. Mass Spectrometry in the Clinical Laboratory — Challenges for Quality Assurance. Spectrosc Suppl. 2015;13(3).

[39] Tarabichi S, Abe EA, Lizcano JD, Parvizi J. New diagnostic tools for the diagnosis of periprosthetic joint infection. In: Ruggieri P, Parwani R, Emara KM, Angelini A, editors. Bone and joint infections. Springer Cham; 2025. p. 69–75.

[40] Kverneland AH, Harking F, Vej-Nielsen JM, Huusfeldt M, Bekker-Jensen DB, Svane IM, et al. Fully Automated Workflow for Integrated Sample Digestion and Evotip Loading Enabling High-Throughput Clinical Proteomics. Mol Cell Proteomics. 2024;23(7, 100790):1–11. 10.1016/j.mcpro.2024.100790 PMC11251069 38777088

[41] Hayrapetyan H, Tran T, Tellez-corrales E, Madiraju C. Enzyme-linked immunosorbent assay: types and applications. In: Matson RS, editor. ELISA methods in molecular biology. New York, NY: Humana; 2023. p. 1–17. 10.1007/978-1-0716-2903-1_1 36795355

[42] Wouthuyzen-Bakker M, Ploegmakers JJW, Kampinga GA, Wagenmakers-Huizenga L, Jutte PC, Muller Kobold AC. Synovial calprotectin. Bone Jt JournalJoint J. 2017;99B(5):660–5. 10.1302/0301-620X.99B5.BJJ-2016-0913.R2 28455476

[43] Grzelecki D, Walczak P, Szostek M, Grajek A, Rak S, Kowalczewski J. Blood and synovial fluid calprotectin as biomarkers to diagnose chronic hip and knee periprosthetic joint infections. Bone Joint J. 2021;103-B(1):46–55. 33380202 10.1302/0301-620X.103B1.BJJ-2020-0953.R1

[44] Alkadhem MF, Jutte PC, Wouthuyzen-Bakker M, Muller Kobold AC. Analytical and clinical considerations of synovial fluid calprotectin in diagnosing periprosthetic joint infections. Crit Rev Clin Lab Sci. 2025;62(3):228–39. 39969081 10.1080/10408363.2025.2463634

[45] Hoffman AP, Grzelecki D, Abdeen A, Kocaoglu H, Abdelnasser MK, Alex T, et al. 2025 ICM : Synovial Analysis. J Arthroplasty. 2025;0(0):1–10. 10.1016/j.arth.2025.10.080 41176116

[46] Matsen Ko L, Parvizi J. Diagnosis of Periprosthetic Infection. Novel Developments. Orthop Clin North Am. 2016;47(1):1–9. 10.1016/j.ocl.2015.08.003 26614915

[47] Zahar A, Lausmann C, Cavalheiro C, Dhamangaonkar AC, Bonanzinga T, Gehrke T, et al. How reliable is the cell count analysis in the diagnosis of prosthetic joint infection? J Arthroplasty. 2018;33(10):3257–62. 29887359 10.1016/j.arth.2018.05.018

[48] Kung MS, Markantonis J, Nelson SD, Campbell P. The synovial lining and synovial fluid properties after joint arthroplasty. Lubricants. 2015;3(2):394–412.

[49] Mi P, Dong H, Chen S, Gao X, Cao X, Liu Y, et al. Association between HDL-C and chronic pain: data from the NHANES database 2003–2004. Front Med. 2024;11(1340037):1–9. 10.3389/fmed.2024.1340037 PMC10961440 38529119

[50] Sorić Hosman I, Kos I, Lamot L. Serum Amyloid A in Inflammatory Rheumatic Diseases: A Compendious Review of a Renowned Biomarker. Front Immunol. 2021;11(631299):1–27. 10.3389/fimmu.2020.631299 PMC7933664 33679725

[51] Zhang X, Lu F, Geng B, Zhang B, Guo L, Xia Y. Serum amyloid A is a promising tool for screening periprosthetic joint infection. Clin Orthop Relat Res. 2025;483(12):2308–17. 40388800 10.1097/CORR.0000000000003547 PMC12629361

[52] Hodges NA, Sussman EM, Stegemann JP. Aseptic and septic prosthetic joint loosening: impact of biomaterial wear on immune cell function, inflammation, and infection. Biomaterials. 2021;278:1–10. 10.1016/j.biomaterials.2021.121127 34564034

[53] Panez-Toro I, Heymann D, Gouin F, Amiaud J, Heymann MF, Córdova LA. Roles of inflammatory cell infiltrate in periprosthetic osteolysis. Front Immunol. 2023;14(1310262):1–15. 10.3389/fimmu.2023.1310262 PMC10722268 38106424

[54] Salive ME. Multimorbidity in older adults. Epidemiol Rev. 2013;35(1):75–83. 23372025 10.1093/epirev/mxs009

[55] Fabbri E, An Y, Zoli M, Simonsick EM, Guralnik JM, Bandinelli S, et al. Aging and the burden of multimorbidity: associations with inflammatory and anabolic hormonal biomarkers. J Gerontol A Biol Sci Med Sci. 2015;70(1):63–70. 25104822 10.1093/gerona/glu127 PMC4296167

[56] Pupaibool J, Tarabichi S, Shahi A, Linton A, Abdelnasser MK, Abdelbary H, et al. 2025 ICM : Serological Diagnosis of Surgical Site Infection ( SSI )/ Periprosthetic Joint Infection ( PJI ). J Arthroplasty. 2025;0(0):1–13. 10.1016/j.arth.2025.10.102 41176106

[57] Gabay C, Kushner I. Acute-Phase Proteins and Other Systemic Responses to Inflammation. N Engl J Med. 1999;340(6):448–54. 9971870 10.1056/NEJM199902113400607

[58] Goswami K, Parvizi J, Maxwell CP. Current Recommendations for the Diagnosis of Acute and Chronic PJI for Hip and Knee—Cell Counts, Alpha-Defensin, Leukocyte Esterase. Next-generation Sequencing Curr Rev Musculoskelet Med. 2018;1(11):428–38. 10.1007/s12178-018-9513-0 PMC6105482 30062484

