# Supplementary material for: Calprotectin identified as a key differentially expressed plasma biomarker in periprosthetic joint infection against aseptic failure: a precision health proteomics study
Source: Arthroplasty. 2026 Sep 4;8:67. doi: 10.1186/s42836-026-00414-6 (PMC13543562; doi:10.1186/s42836-026-00414-6)
Supplement: Supplementary file 1 [file 42836_2026_414_MOESM1_ESM.docx]

**Supplementary**

**Fig. S1.** Volcano plot showing differentially abundant proteins between test conditions, Chronic pain vs. Aseptic failure. Gray points represent differentially abundant genes with no significance. A positive difference indicates the upregulated proteins of the first comparison group in the subheading against the second comparison group, whereas a negative difference indicates the downregulated proteins of the first comparison group in the subheading against the second comparison group.

**Fig. S2.** Functional enrichment analysis (A–D) of significantly upregulated (A&C) and downregulated (B&D) regulated proteins in Infection vs Sterile, sorted by FDR.

**Fig. S3.** Volcano plot showing differentially abundant proteins between test conditions with no imputation applied to missing protein intensity values: (**A**) Infection vs Aseptic failure and (**B**) Infection vs Chronic pain. Gray points represent differentially abundant genes with no significance. A positive difference indicates the upregulated proteins of the first comparison group in the subheading against the second comparison group, whereas a negative difference indicates the downregulated proteins of the first comparison group in the subheading against the second comparison group.

**Fig. S4.** Volcano plot showing differentially abundant proteins between test conditions with or without patients with rheumatoid disease and/or biological immunotherapy. Imputation applied to missing protein intensity values (**A, C, E**), Infection vs Aseptic failure, and (**B, D, F**) Infection vs Chronic pain. Patients with Rheumatoid Disease were excluded (**A, B**). Patients taking biological immunotherapy were excluded (**C, D**). Patients with both Rheumatoid disease and taking biological immunotherapy were excluded (**E, F**). Gray points represent differentially abundant genes with no significance. A positive difference indicates the upregulated proteins of the first comparison group in the subheading against the second comparison group, whereas a negative difference indicates the downregulated proteins of the first comparison group in the subheading against the second comparison group.

**Supplemental Table S1.** Linear model adjusting for rheumatoid disease/inflammatory condition and biological immunotherapy.

| **Linear model** | **Comparison** | **Estimated ratio** | **95% CI** | ***p*-value** |
| --- | --- | --- | --- | --- |
| Unadjusted | Aseptic failure vs Infection | 0.567 | 0.415–0.775 | **0.00054** |
|  | Chronic Pain vs. Infection | 0.562 | 0.387–0.817 | 0.00296 |
| Adjusted | Aseptic failure vs Infection | 0.574 | 0.419–0.787 | **0.00078** |
|  | Chronic Pain vs. Infection | 0.568 | 0.388–0.831 | 0.00414 |
|  | Rheumatoid disease / inflammatory condition, yes vs no | 0.908 | 0.536–1.537 | 0.305 |
|  | Biological immunotherapy, yes vs no | 1.31 | 0.778–2.206 | 0.715 |

One patient was removed in both models due to NA in Rheumatoid disease/inflammatory condition. *p*-values in bold indicate a value ≤ 0.001.
